# Genome Sequence of the Attenuated Carbosap Vaccine Strain of *Bacillus anthracis*

**DOI:** 10.1128/genomeA.00067-12

**Published:** 2013-01-15

**Authors:** Robin Harrington, Brian D. Ondov, Diana Radune, Mary Beth Friss, Joy Klubnik, Lynn Diviak, Jonathan Hnath, Stephen R. Cendrowski, Thomas E. Blank, David Karaolis, Arthur M. Friedlander, James P. Burans, M. J. Rosovitz, Todd Treangen, Adam M. Phillippy, Nicholas H. Bergman

**Affiliations:** a National Biodefense Analysis and Countermeasures Center, Frederick, Maryland, USA; b United States Army Medical Research Institute for Infectious Diseases, Frederick, Maryland, USA

## Abstract

The *Bacillus anthracis* Carbosap genome, which includes the pXO1 and pXO2 plasmids, has been shown to encode the major *B. anthracis* virulence factors, yet this strain’s attenuation has not yet been explained. Here we report the draft genome sequence of this strain, and a comparison to fully virulent *B. anthracis*.

## GENOME ANNOUNCEMENT

*Bacillus anthracis* is a Gram-positive, spore-forming, filamentous bacillus and is the causative agent of anthrax in both animals and humans (1). *B. anthracis* strains attenuated for virulence in animals that were described in the latter part of the 19th century, and in particular, those put forward by Pasteur, soon came into use for vaccination against anthrax in cattle and sheep (2).

Many attenuated strains of *B. anthracis* lack one of the two virulence-associated plasmids pXO1 and pXO2. The Sterne-type strains lack pXO2 and are toxigenic but cannot produce a capsule, while the Pasteur-type vaccine strains lack the pXO1 plasmid and are therefore encapsulated but nontoxigenic. Less commonly, other strains have been shown to be attenuated despite retaining both the pXO1 and pXO2 plasmids, which would normally result in full virulence (3, 4).

One example of such a strain is *B. anthracis* Carbosap, used in Italy as a live spore vaccine for cattle and sheep (5). The Carbosap genome has been previously shown to contain sequences that indicate the presence of pXO1 and pXO2 as well as a limited set of chromosomal markers associated with virulence, and yet it shows significant attenuation of virulence in rabbits (5, 6, 7).

In order to define the differences between Carbosap and other strains of *B. anthracis* that may explain its attenuation, we sequenced the genome of Carbosap. Genomic DNA was prepared from the Carbosap strain of *B. anthracis* (obtained from A. Fasanella) using standard methods (8) and sequenced using the 454 and Illumina MiSeq platforms. A total of 344,312 454 reads and 1,719,544 Illumina reads, totaling 390 Mb, were assembled using Newbler version 2.7 in both *de novo* and reference modes, using *B. anthracis* strain Ames Ancestor as a reference (9). Contigs from the *de novo* assembly that showed structural variation with respect to the reference were combined with the reference assembly using Minimus 2 (part of AMOS 3.1.0) to produce a draft genome of 5,402,970 bp and 21 contigs.

Whole-genome single-nucleotide polymorphism (SNP) analysis showed that the Carbosap strain is a member of the trans-Eurasian clade of *B. anthracis*; the genome also contains 3 chromosomal deletions of 29, 24, and 3.5 kb relative to virulent strains of *B. anthracis*, consistent with previous array and PCR analyses (10). There were no major changes found in the Carbosap pXO1 and pXO2 sequences relative to plasmids from the Ames Ancestor strain. The chromosomal deletions contain >50 annotated genes, many of which have a known or proposed function that could be linked to virulence, so the precise nature of Carbosap’s attenuation remains unclear. A similar situation was recently reported for the *B. anthracis* CDC 684 strain (11), and it seems likely that in both cases further investigation into the functional effects of the genomic changes in these two strains will yield new insights into *B. anthracis* pathogenesis and aid in the identification of potential targets for development of new anthrax therapeutics.

### Nucleotide sequence accession number. 

This Whole Genome Shotgun project has been deposited at DDBJ/EMBL/GenBank under the accession no. ANAO00000000. The version described in this paper is the first version.
